# Impact of the 2008 Economic and Financial Crisis on Child Health: A Systematic Review

**DOI:** 10.3390/ijerph110606528

**Published:** 2014-06-23

**Authors:** Luis Rajmil, María-José Fernandez de Sanmamed, Imti Choonara, Tomas Faresjö, Anders Hjern, Anita L. Kozyrskyj, Patricia J. Lucas, Hein Raat, Louise Séguin, Nick Spencer, David Taylor-Robinson

**Affiliations:** 1 Agència de Qualitat i Avaluació Sanitàries (AQuAS), Barcelona 08005, Spain; 2 Fundació Institut Mar d’Investigacions Mèdiques (IMIM), Barcelona 08003, Spain; 3 CIBER de Epidemiología y Salud Pública, Madrid 28029, Spain; 4 General Practitioner, Homer 22, Barcelona 08023, Spain; E-Mail: 14848mfs@comb.cat; 5 Academic Division of Child Health, The Medical School, University of Nottingham, Derbyshire Children’s Hospital, Uttoxeter Road, Derby DE22 3DT, UK; E-Mail: Imti.Choonara@nottingham.ac.uk; 6 Division of Community Medicine, Department of Medical and Health Sciences, Linköping University, Linköping S-581 85, Sweden; E-Mail: tomas.faresjo@liu.se; 7 Centre for Health Equity Studies (CHESS), Karolinska Institutet, Stockholm University, Stockholm 106 91, Sweden; E-Mail: anders.hjern@chess.su.se; 8 Department of Pediatrics; University of Alberta, University Ave., Edmonton, AB 11402, Canada; E-Mail: kozyrsky@ualberta.ca; 9 School for Policy Studies, University of Bristol, Bristol BS8 1TZ, UK; E-Mail: Patricia.Lucas@bristol.ac.uk; 10 Department of Public Health, The Erasmus University Medical Center, Rotterdam, Dr. Molewaterplein 50, P.O. Box 2040, 3000 CA Rotterdam, The Netherlands; E-Mail: h.raat@erasmusmc.nl; 11 Department of Social and Preventive Medicine, Université de Montréal, C.P. 6128, Succursale Centre-ville, Montréal (Québec), QC H3C 3J7, Canada; E-Mail: louise.seguin@umontreal.ca; 12 Division of Mental Health and Wellbeing, Warwick Medical School, University of Warwick, Coventry CV4 7AL, UK; E-Mail: n.j.spencer@warwick.ac.uk; 13 Department of Public Health and Policy, University of Liverpool, Liverpool L69 3GB, UK; E-Mail: David.Taylor-Robinson@liverpool.ac.uk

**Keywords:** adolescent, child health, economic and financial crisis, inequalities

## Abstract

The aim of this study was to provide an overview of studies in which the impact of the 2008 economic crisis on child health was reported. Structured searches of PubMed, and ISI Web of Knowledge, were conducted. Quantitative and qualitative studies reporting health outcomes on children, published since 2007 and related to the 2008 economic crisis were included. Two reviewers independently assessed studies for inclusion. Data were synthesised as a narrative review. Five hundred and six titles and abstracts were reviewed, from which 22 studies were included. The risk of bias for quantitative studies was mixed while qualitative studies showed low risk of bias. An excess of 28,000–50,000 infant deaths in 2009 was estimated in sub-Saharan African countries, and increased infant mortality in Greece was reported. Increased price of foods was related to worsening nutrition habits in disadvantaged families worldwide. An increase in violence against children was reported in the U.S., and inequalities in health-related quality of life appeared in some countries. Most studies suggest that the economic crisis has harmed children’s health, and disproportionately affected the most vulnerable groups. There is an urgent need for further studies to monitor the child health effects of the global recession and to inform appropriate public policy responses.

## 1. Introduction

The current global economic and financial crisis, which began at the end of 2007 in the U.S., poses a major threat to health and affects mainly Europe and several other countries [1]. Economic downturns are known to affect health and living conditions of the populations. The impact of crisis in each country depends on the type of recession (duration and intensity, the speed of changes, and the types of changes that occur), the situation prior to the recession, the measures adopted by the states and governments in response to the crisis, and the role played by communities and family structure in the lives of individuals [2]. 

There is a large body of work on recession and health, most of it in adults. There are both positive (*i.e.*, road traffic accidents go down) and negative effects (*i.e.*, suicides generally go up) documented [3]. There are fewer data for children and youth. Children are a vulnerable population group and strong evidence exists on the link between socioeconomic living conditions and child health [4]. The literature on previous recessions in different countries and periods suggest that exposure to poverty in early life and during childhood for prolonged periods may have a strong and irreversible impact on physical, cognitive and social health of the childhood population [5]. Exposure to poverty in early life has been shown to be associated with a higher risk of chronic health conditions of elderly people such as cardio-vascular diseases [6,7] and Alzheimer’s disease [8,9,10].

The role of social determinants of health is essential in the pathways of influence of economic crisis on child health [11]. Reduced opportunities for employment increases income poverty, restrict food budget, decrease housing security/quality (e.g., via evictions and moves) and harm parental mental health [12]. Increased food costs restrict food budgets for all. Decreased spending on public services could lead to reduced health care provision, decreased social protection spending, and cuts in effective health promotion initiatives, including maternal health and early child development programmes. Child labour may increase with attendant impacts on health and education [5,13].

The level of social inequalities and social gradients during childhood in itself can also have an important role on health outcomes both in the short and the longer term [14]. Mechanisms of social protection (such as social welfare payments) implemented by countries are likely to be effective in mitigating the effects of economic shocks on child health [15], but austerity measures adopted in many countries during the current economic crisis have reduced social protection mechanisms, thus potentially contributing to increased health inequalities [1,3]. Some studies have reported on the impact of the current economic crisis on families and children in Greece [16], Spain [17,18], the UK [19], and the U.S. [20]. In Greece youth unemployment rose from 18.6% to 40.1% from 2008 to 2011. In Spain, child poverty increased by 53% between 2007 and 2010. There are an estimated 3.5 million children living in poverty in the UK and this figure is expected to increase by 600,000 by 2015/2016 [21]. However, few data exist on the impact of the current economic crisis on child health so it is important to summarise what we know across studies reporting to date.

The objective of the present systematic review is to provide an overview of studies in which the impact of the 2008 economic and financial crisis on child health was analysed, in order to assess the evidence base to inform public health policy and identify research gaps.

## 2. Methods

A structured search was carried out in September 2013 in the databases PubMed, and ISI Web of Knowledge using terms for financial recession and terms for childhood. Languages included Dutch, English, French, Italian, Spanish, and Swedish. A Google search using the terms “crises OR downturn OR recession OR Economic Recession OR austerity AND child OR adolescent OR youth OR infant OR paediatrics” was also conducted, and the first 200 entries from Google were screened. All references lists of included studies were included, and an expert group, the International Network for Research in Inequalities in Child Health, were asked to identify any additional studies known to them.

### 2.1. Inclusion and Exclusion Criteria

Both quantitative and qualitative studies were included, where they considered the impact of the current economic crisis on child health (children and young people <18 year). Longitudinal studies needed to compare a period before and after December 2007 (starting point of the current crisis), cross sectional studies needed to have collected data during the period 2008–2013. General population studies were included if results on the child population were identified separately. Outcomes included any mortalities, morbidities or wider child health impacts. Intermediate outcomes such as food consumption patterns were also considered given their potential for short and long term impacts on child nutrition and physical health. Studies on the impact of previous economic crises were excluded in the analysis as well as studies only analysing adult health. Given the scarcity of data, grey literature and none peer reviewed manuscripts were included if other selection criteria were fulfilled.

### 2.2. Study Selection

Abstracts obtained by the initial search strategy were assessed for possible inclusion by two researchers (L. Rajmil, M.J. Fernández de Sanmamed) and full text papers of all studies potentially includable (or unclear). Differences of opinion on inclusion were resolved by consensus.

### 2.3. Study Appraisal

The risk of bias of included studies was assessed by two authors (L. Rajmil, M.J. Fernández de Sanmamed) using the Strengthening the Reporting of Observational Studies in Epidemiology (STROBE) initiative for quantitative studies [22]. Qualitative studies were assessed using EPICURE (Engagement, Processing, Interpretation, Critique Usefulness, Relevance and Ethics) [23]. For quantitative studies, each criterion, from the total of 22, was awarded one point, 0.5 or 0 (Zero) points if adjudged to be fully, partially or not at all presented; studies scoring 16 or over were adjudged to have a low risk of bias; those scoring 7–15 as moderate risk, and those scoring <7 as high risk of bias. For qualitative studies, four out of the seven items (P, I, U, R) were considered essential to assess studies as average or low risk of bias (the latter if also met at least one of the other items). The lack of one or more of these four essential items was considered as high risk of bias. Given the scarcity of data on the study subject it was decided to avoid using quality assessment criteria as an exclusion criteria but to further carry out a sensitivity analysis and to assess the strengths of evidence of the included studies.

### 2.4. Data Extraction and Analysis

Data were extracted using a standardised data extraction form. Data extracted included: setting (according to the country: international, national or regional study); type of study (before-after comparative study, cross-sectional study, *etc.*); objectives of the study; years covered by the study; the specific target population; age range; exposure(s) measure(s) investigated (individual or ecologic variables such as unemployment rate, Gross Domestic Product, sociodemographic variables); outcome measures (classified as mortality, health-related quality of life, mental health, *etc.*); and the results in terms of impact on child health. The data was classified and organised according to the main outcome measure of the study. Quantitative and qualitative studies were analysed separately and the results presented together according to the outcome measure. Given the heterogeneity of studies in terms of study design (mainly observational and descriptive), participants, and outcomes, we undertook a narrative synthesis of the results. These are reported in the following categories: infant and child mortality; food habits and nutrition; health behaviours, non-accidental injuries, mental health and health-related quality of life; and chronic conditions.

## 3. Results

**Figure 1:**
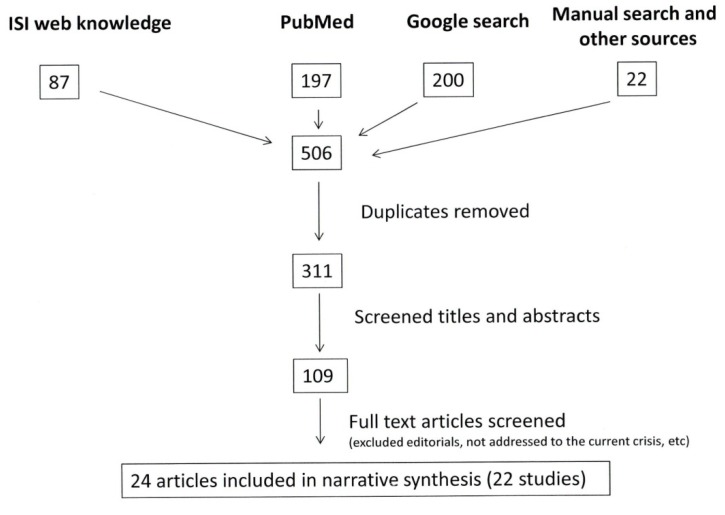
Flow chart of the study selection process.

Figure 1 shows the results of the literature search. In all 506 documents were screened. From the 311 titles and abstracts reviewed 200 were initially excluded, most of them addressing previous crises and/or the impact on adult health; 109 documents were full text screened, and finally 24 documents corresponding to 22 studies were included (one study published three documents).

### 3.1. Study Characteristics

Nineteen quantitative studies, two qualitative studies and one study with mixed quantitative and qualitative methods were included in the review. Thirteen quantitative studies analysed trends over time [24,25,26,27,28,29,30,31,32,33,34,35,36] and four of them showed a low risk of bias (Table 1) [25,31,32,36]. Six were cross-sectional studies [37,38,39,40,41,42] three of which showed a low risk of bias [38,39,41]. Two qualitative studies were included: one was carried out in developing countries [43,44,45], and one in the UK [46]. All except one qualitative sub-study [45] showed low risk of bias on the EPICURE assessment. The study with mixed methods [47] showed moderate risk of bias on its quantitative approach and low risk of bias on qualitative approach.

**Table 1:** Characteristics of included studies.

First Author	Countries	Type of Study	Year(s)	Population/Sample	Source of Data (n)	Outcome Measure (s)	Risk of Bias (Score) "
**Quantitative studies: trends over time**							
Ariizumi [24]	Canada	Mortality trends	1977–2009	All ages	Canadian Vital Statistics Data	Under 15 year mortality	15
Friedman [25]	30 Sub-Saharan African countries	Infant mortality trend modelisation	2007–2009	<1 year /Women 15–49 years old	Demographic Health Survey. Data from 260,000 women and 640,000 live births	Infant mortality	16
Simó [26]	Greece, Spain, OECD "" countries	Infant, neonatal, and perinatal mortality trends	1990–2010	<1 year	OECD "" data on mortality	Infant, perinatal and neonatal	7.5
Vlachadis [27]	Greece	Descriptive time-trends	1966–2010	All recorded live births and fetal deaths	Helenic Statistical Authority	Stillbirths per 1000 women	7.5
Gordon [28]	UK	Cross-sectional survey and time trends description	Surveys conducted in 1982, 1990, 1999, 2012	Children (ages not specified)	Necessities of Life survey (N = 2462 adults). Living standard survey (N = 5193 households)	Basic needs (30 items for children). Multiple deprivation	14.5
Department for Environment Food and Rural Affairs (DEFRA) [29]	UK	Trends based on cross-sectional repeated surveys	2007– 2011	General population of households with children (ages not specified)	Family Food Survey (N = approximately 6000 households a year)	Household spending on food, and comparisons with healthy diet	12.5
Sulaiman [30]	Bangladesh	Longitudinal rural panel and 2 urban cross sectional samples	2006, 2008	Children 0–59 months in 2006/24–82 months in 2008	Nutritional project 2006. In 2008: subsample of 1163 rural households, and 435 urban households	Weight-for-height Z score in children. Changes of food consumption.	12.5
Berger [31]	U.S.	Comparison before-during the recession	2004–2007/December 2007–June 2009	<5 year Population of 74 counties in 3 areas.	Incidence of AHT ^‡^ in children under 5 year. Hospital discharge records (N = 422)	Changes in rates of hospitalisation due to AHT	20
Huang [32]	OH, U.S.	Incidence rates over time	2001–November 2007/December 2007–June 2010	<2 year	Incidence of injuries and AHT ^‡^. Hospital discharge records (N = 639). Study from 1 hospital	Changes in rates of hospitalisation due to AHT ^‡^	18
Brooks-Gunn [33]	U.S.	Prospective cohort study	May 2007–February 2010	9 year-old children	Wave of Fragile Families Child Well-being Study. 5000 families of 20 large cities from 15 States	Frequency of maternal spanking	16
Cui [34]	U.S.	Trends based on repeated cross-sectional surveys	2001–2010 U.S. general population sample	12–17 year	National Health and Nutrition Examination Survey (NHAMES)	Self-rated health, unhealthy days and activity limitation days	16
Millet [35]	U.S.	Before-after approach	2001–2010	Child under 18 year	Public available administrative data for 7 States	Child abuse and maltreatment reported to social services	10
Rajmil [36]	Catalonia, Spain	Before-after approach	2006–2010/2012	0–14 year General population	Catalan Health interview Survey 2006 (N = 2200) and Continuous health survey 2010–2012 (N = 1967)	Health behaviors, obesity, mental health and perceived heatlh and quality of life	17
**Quantitative studies: cross sectional**							
Catalonian ombudsman [37]	Catalonia, Spain	Cross-sectional	2013	<16 year General population from Catalonia	European Union Statistics on Income and Living Conditions (EU-SILC) and other sources	Reported food consumption. Multiple deprivation	5.5
Samuels [47]	Nigeria	Cross sectional	2009	Households with children	National household surveys	Reported food consumption	10
Bruening [38]	MN, U.S.	Cross-sectional	2009–2010 Population-based study	Adolescents	Household Food Insecurity questionnaire (Families and Eating Activity Among Teens study) (N = 2095 parents)	Family food security status	17
Jackson [39]	U.S.	Comparison of two cross-sectional studies	2008–2010	<18 year children with asthma and their parents	Behavioural Risk Factor Surveillance System phone interview (2008 N = 4133/2010 N =3492 parents)	Parental smoking behavior	16
Pearlman [40]	U.S.	Cross-sectional	2007–2009	2–17 year children with asthma	U.S. national Child Asthma Call-Back survey. Parents of children with asthma (N = 5138)	Level of asthma control	13.5
Tarantino [41]	U.S.	Cross-sectional survey	2009–2010	<26 year Patients with haemophilia and their caregivers (where <26 year, average age = 11.2 year) Hematologists	Survey designed *ad hoc* (N = 70 caregivers of <26 year with Hemophilia A, and adult patients, N = 64)	Changes in treatment decision-making after downturn. Attitude towards healthcare reform	16
Anagnostopoulos [42]	Greece	Descriptive cross sectional study	2000–2011	Child and adolescent population attending psychiatric services	National Action Plan *Psychargos* on mental healthcare services	Changes on the distribution of mental health diagnoses among users	6
**Qualitative studies**							
Heltberg [43] Hossain [44] Hossain [45]	Developing countries 17 in 2008, 6 in 2009, 4 in 2011	Focus groups and interviews	2008–2011	Children (ages not specified. Respondents selected that represent groups exposed to economic shock)	To study perceptions and behaviors of people as live crisis impact and major coping responses used by poor and vulnerable people and households		EPICURe, EPICURe and epicURe respectively
Samuels [47]	Nigeria	Key informants interviews, focus groups and in-depth interviews	2009	Children (ages not specified) 6 Nigerian zones that reflect demographic and socioeconomic heterogeneity	To analyse the impact of 3F ^†^ crisis on vulnerable social groups (women and children) and coping strategies undertaken by households during the period of food, fuel and financial crisis		EPICURe
Halls [46]	UK	Semi-structured interviews and participant observation (Ethnographic approach)	December 2011 and January 2012	Theoretically driven sample of 11 families with children (ages not specified) with different social status over 5 visits	To analyse the lived experience of families against a backdrop of austerity. Impact of austerity on family life and family food choices		ePIcURE

Notes: * The risk of bias was assessed using the STROBE criteria for quantitative studies (range 0–22 points); for qualitative studies the uppercase and lowercase indicate compliance or non-compliance (or absence) of a given item from the 7 items of the (see methods section for more information); ** OECD: Organisation for Economic Cooperation and Development; **^‡^** AHT Abussive head trauma; **^†^** 3F crisis: food, fuel and financial shock.

#### 3.1.1. Infant and Child Mortality

Four studies reported mortality outcomes. One modelling study estimated an excess of 28,000–50,000 infant deaths in the year 2009 in sub-Saharan African countries based on interviews with a random sample of 260,000 mothers (Table 2) [25]. National vital statistics in Greece reported an increase of 32% in stillbirths between 2008 and 2010, and also increases in infant, perinatal and neonatal mortality [27], while no influence was found on infant mortality in Spain [26], or under 15 year mortality in Canada [24].

**Table 2:** Impact of the 2008 economic crisis on infant and child mortality.

Countries	Results	Study
30 Sub Saharan Africa Countries	Excess of 28,000–50,000 infant deaths in 2009, most of them girls (compared to period 1977–2008). There are 3 million infant deaths a year in these countries (infant mortality rate was 90/1000 in 2005).	Friedman J. [25]
Greece, Spain	Perinatal, neonatal and infant mortality all increased by 20% to 30% from 2008 to 2010 in Greece. No changes were found for Spain.	Simó J. [26]
Greece	Stillbirth rate increased from 3.31/1000 in 2008 to 4.28/1000 in 2009 and 4.36/1000 in 2010 (a 32% increase over 2 years).	Vlachadis N. [27]
Canada	No influence of parental unemployment on mortality in children under 15 year when comparing the period 1977–2008 to 2009.	Ariizumi H. [24]

#### 3.1.2. Food Habits and Nutrition

Four quantitative studies reported on the food habits and one on nutritional status, and there were three qualitative studies exploring food habits and nutrition (Table 3). Studies in Europe (Spain [37] and UK [29]), U.S. [38] and Bangladesh [30] all demonstrated a significant adverse effect of the economic crisis on food intake by children, and specifically in vulnerable children. In particular the study in the UK by DEFRA [29] showed a social gradient with children from low income families eating less fruit and vegetables between 2007 and 2011. A study in Bangladesh showed an increase in the number of children who were underweight [30].

Qualitative studies found similar and consistent results both in developed and developing countries [43,44,45,46,47]. The increase in food prices was associated to diminishing the number and quality of meals and buying cheaper food. A gender effect was noted since the results showed that more women were affected by the crisis compared to men. Poor children from urban areas in developing countries were affected the most [47]. The usual strategy followed by families was shown to be to reducing costs by giving foods based more on carbohydrates in developed and developing countries and fast food offered an easy solution according to the UK study [46].

#### 3.1.3. Health Behaviours, Non-accidental Injuries, Mental Health and Health-related Quality of Life

Twelve studies reported on health behaviours, child maltreatment, mental health and health-related quality of life (see Table 4). Higher probability of smoking for unemployed parents in families having children with asthma was found in the U.S. in 2010, but this association was not found in 2008 [39].

**Table 3:** Impact of the 2008economic crisis on food and nutrition.

Country	Results	Study
***Quantitative Findings***		
U.S.	39% of parents experienced food insecurity in 2009/2010 and 13% had very low food security. Food insecure parents were more likely to be non-white, single parent, low education level, unemployed and with low income.	Bruening M. [38]
UK	In 2012 500,000 children (4%) in the UK live in families who cannot afford to feed them properly.	Gordon D. [28]
UK	Households purchased 4.2% less food in 2011 than in 2007 while spending 12% more. Low income decile households have bought less fruits and vegetables.	Department for Environment Food and Rural Affairs (DEFRA) [29]
Spain	9.8% of families with children under the age of 16 years could not afford regular fish or meat on alternate days. This figure had risen from 1.7% in 2008.	Catalonian Ombudsman [37]
Bangladesh	The weight-for-height Z score in 2008 was below the trend line for >30–59 months old children at baseline. More affected groups were 30–59 months and 0-6 months old. In the rural sample wasting and underweight was 5.5%, and this figure was 6.7% for the urban sample comparing with 2006.	Sulaiman M. [30]
***Qualitative Findings***		
UK	Food was often the first area to be cut in the family budget. Families get this buying in cheaper supermarkets, less fresh fruit and vegetables and more frozen. Trading down was a common strategy lowering the quality. Another way to reduce costs for families on lower incomes is buying food that would fill children up (more rice or pasta or samosas with chips). Fast food offers also an easy solution.	Halls S. [46]
Data from 17 developing countries	Food insecurity emerged as the most severe impact of the crisis. It was more pronounced in Central African Republic and Kenya, and was also common in Bangladesh and Zambia. Reducing the quality of food and the number of meals was the most common behaviour-based coping response to crises. Problem of sending children to school on an empty stomach was widely cited.	Heltberg R. [43] Hossain N. [44] Hossain N. [45]
Nigeria	Families reduced consumption of food in terms of quality and quantity in Nigeria. Urban poor are more adversely affected because the rural poor have their own food production. Informants identified children and women from poor families as bearing the brunt of rising costs of food.	Samuels F. [47]

**Table 4:** Summary of the impact of the 2008 economic crisis on health behaviors, non-accidental injuries, mental health and health-related quality of life.

Country	Results	Study
***Quantitative Findings***		
U.S.	Comparing 2007 and 2010, unemployed parents showed higher probabilities to be current smokers only in 2010 OR = 1.8 (1.24–2.61). Lower level of education, and mental distress were other associated factors in both surveys.	Jackson T.L. [39]
U.S.	Increasing incidence rates of AHT " was observed from 8.9/100,000 in 2004–2007 to 14.7/100,000 in 2007–2010 (1.30 to 1.7) for AHT " during the recession period in 3 different areas from the U.S. compared to the previous period. No association was found on this outcome when adujusting for unemployment rates.	Berger R.P. [31]
U.S.	Increasing incidence rates on AHT ", from 0.7/month to 1.4/month from the non-recession to the recession period. Unemployment rates in Ohio have risen after the increase in AHT.	Huang M.I. [32]
U.S.	A Relative risk = 1.06 (*p* < 0.05) was found for high frequency of spanking in periods of worse consumarer confidence (Consumer Sentiment Index). No significant changes were observed when taking into account the rest of variables in the model (unemployment rates and home foreclosure).	Brooks-Gunn J. [33]
U.S.	No clear association was found between pre-recession and post recession period on different types of child maltreatment measures in seven U.S. States.	Millet L. [35]
Greece	Prevalence of psychosocial problems have risen by 40%, conduct disorders by 28%, school leaves by 25%, bullying by 22%, suicide attempts 20%, illegal and additive substances have risen by 19%, and family conflicts by 51%.	Anagnostopoulos D.C. [42]
U.S.	Self-rated health and reported mental health declined significantly, specially among adolescents in low-income families, at the end of the decade analyzed.	Cui W. [34]
Spain	A mixture impact on health was found: some health behaviors have improved as an average in the whole population (*i.e.*, time spent on screen) while inequalities in health-related quality of life appeared according to the level of education, inequalities in mental health remained, and obesity showed an important increase.	Rajmil L. [36]
UK	The number of children who are multiple deprivated (at least two basic needs uncovered regarding food, clothing crowded houses, and social participation) was 2 million in 1999 and 4 million in 2012. Multiple-deprived housesholds in Britain have increased from 14% in 1983 to 33% in 2012.	Gordon D. [31]
Spain	Families suffering severe material deprivation (at least 4 items from a list of minimum 9 items) was 5.8% in 2011 and 1.6% in 2008.	Ombudsman C. [37]
***Qualitative Findings***		
17 developing countries	Stressed women took their frustrations out on children. Children were often left alone as all adult family members had to work long hours away from home. Tensions within households we fairly clear and well-established, even in absence of domestic violence or abuse. Criminalisation and/or substance abuse among young people was directly attributed to the pressure of the crisis. In some countries young women, and even men, were reported to be increasingly entering sex work (Kenya, Zambia, Dhaka, Lusaka, Nairobi).	Heltberg R. [43] Hossain N. [44] Hossain N. [45]
Nigeria	Mothers reported worsening mental health and changes in the amount of child care and protection. Children are adopting harmful coping mechanismes like illegal livelihoods, sex work, early marriage of girls, exploitative child labour, distress sale.	Samuels F. [47]

Note: * AHT: abussive head trauma.

An increase in the incidence rates in the U.S. for abusive head trauma (AHT) and child maltreatment was found in three studies [31,32,33], while no influence of the downturn period was found in one study [35]. In Greece, an increase was reported for child and adolescent patients in hospital care for various psychosocial problems from 2007 to 2011 [42].

Self-rated health declined and mentally unhealthy days increased in adolescents from the U.S., mainly among those in low-income families [34], and inequalities in health-related quality of life in children 6–14 year according to maternal level of education appeared after the start of the crisis in Catalonia, Spain [36]. Qualitative studies in developing countries highlight increased family tension and substance abuse among young people attributed to the pressure of the crisis [43,44,45,46,47].

#### 3.1.4. Chronic Conditions

Two studies reported on children with chronic conditions from the U.S. (see Table 5). A gap in health insurance coverage in the previous year was associated to a lower asthma control in children when comparing before and during the current crisis [40].

Fifty four percent of hemophiliac patients or their caregivers reported negative impact on the management of the condition after starting the current crisis [41].

**Table 5:** Summary of the impact of the 2008 economic crisis on chronic conditions.

Country	Results	Study
U.S.	Lack in health insurance coverage (OR = 1.74; 1.07–2.83) was the main factor associated to poorly controlled asthma during the period 2007–2009, together with intermediate-low income level. Higher levels of unemployment were not associated with asthma control.	Pearlman D.N. [40]
U.S.	54% reported negative impact on the management of the Hemophilia in the period 2009/2010 than previously: delayed or cancelled appointments, reduced or skipped doses, skipped filling prescription, delayed bleeding related urgent care visit. 22% anticipated positive influence of healthcare reform*.*	Tarantino M.D. [41]

## 4. Discussion

To our knowledge this is the first review on the effects of the 2008 economic crisis on children’s health. One modelling study estimated a negative impact on infant and child mortality in sub-Saharan countries; and in Greece such impact on mortality was shown by using registered data in another study. Furthermore, quantitative and qualitative studies documented changes in food consumption and nutrition worldwide with specific impact on most vulnerable population. Other studies showed an increase in non-accidental injuries and in social inequalities in perceived health and health-related quality of life in some countries. This review also highlights the gaps in knowledge on the subject and the need for studies to generate sufficient evidence to inform effective measures to mitigate the negative impact of the economic crisis on child health.

A limitation of the review is that most of the studies were not specifically designed to analyse the impact of economic crisis on child health, and are not sufficiently robust to establish a causal relationship. As a result, the level of evidence is weak to establish clear recommendations. Overall the quality of the studies is mixed, with a low likelihood of bias in the studies based on vital statistics or in population representative samples, but lower quality in others. A sensitivity analysis showed that child mortality studies were not of sufficient quality to demonstrate the impact of the crisis on mortality, (e.g., the outcomes estimated on the study from sub-Saharan African countries were based on retrospective data). Qualitative studies consistently showed increased nutritional risk of children of socially disadvantaged families worldwide. An increased risk of child maltreatment has been quite consistent in studies from the U.S., and inequalities in perceived health and quality of life were found in different populations. The rest of the results should be taken with great caution given the high or average risk of bias of the included studies. However, studies were included independently of their risk of bias due to the scarcity of data and to draw attention on the lack of good quality studies. Secondly, some studies present results as averages in the population. Future studies should focus on analysing the subgroups most affected by the crisis. Thirdly, publication bias cannot be ruled out. Fourthly, it should be taken into account that a diverse group of countries—both developed and developing—was included in the review. All these countries did not experience the recession at the same time and did not define it in the same way. As a consequence, some specific results such as infant mortality should not be generalised. Finally, characteristics of the included studies did not allow us to perform a meta-analysis.

Positive effects have been described on adult mortality in previous crises in some specific countries [48]. This effect has not been reported on child mortality either in previous or in the current crisis.

It is worth noting that none of the studies were ideally designed to demonstrate causal relationships. Nevertheless, some of the studies have tried to attribute a causal relationship to the recession making necessary adjustments to strengthen these claims (e.g., adjusting for pre-recession time trends, adjusting for other time varying confounders, among other procedures).

There is sufficient accumulated evidence from previous crises showing that exposure to situations of deprivation and increasing social inequalities are damaging to children's health in the short and long term. Cohort studies of children carried out since the last century have shown the influence of social determinants on physical, cognitive and social health [49,50,51,52], and the negative cumulative effects of deprivation and stress during the first years of life on the physical and mental health status later in childhood [53,54].

High food prices reduce diversity and nutritional quality of the diet, and even quantity [55]. This is likely to impact on the most vulnerable groups in both industrialised and developing countries. The poorest populations from urban areas are the most vulnerable to food insecurity and malnutrition [56]. In developed countries, children living in families without resources or without social protection mechanisms due to austerity measures are at greater nutritional risk, including both obesity and undernutrition. The results of the present review highlight the potential nutritional risk for the most vulnerable populations.

The evidence to date demonstrates the plausibility of the association between the crisis and violence against children. In the adult population, it has been found that mental health problems, family stress, violence and suicides increased in time of crisis [57], and these often occur in families with children.

The included studies do not allow comparison of the measures taken by governments to alleviate the effects of the crisis, and this should be for a focus of future studies. Nevertheless, according to the present data, it cannot be ruled out that those countries adopting austerity measures showed high negative impact on child health, such as southern European countries. Future studies should confirm or discard this fact.

### Implications for Research and Policy

Further research is required to establish the mechanisms by which an economic crisis affects children’s health, and how to measure the exposure. It should be taken into account that economic changes usually occur rapidly, leading to deterioration in the social determinants of health, but consequent changes in health outcomes can have long latency periods and may take decades to become fully apparent.

As currently available indicators are often not sensitive to the short-term impact on health, there is a need to develop indicators that are sensitive to change [15,58].

Cross country studies to assess the effects of the range of responses that have been implemented by governments during the recent economic crisis are needed and should include an assessment of effects on child health. Previous experience, such as the crisis in Nordic countries in the early 90s, suggested that no impact or a minimum impact on children's health occurred in Nordic countries given the protective effect of a highly developed welfare state and additional specific measures of child protection maintained during that crisis [59]. It is also necessary to study the epidemiology of resilience factors in order to determine the factors that mitigate the negative consequences of the crisis, including measures of social capital and family structure [1].

## 5. Conclusions

Most studies suggest that the economic crisis may pose a serious threat to children’s health, and disproportionately affects the most vulnerable groups. There is an urgent need for further studies to monitor the child health effects of the global recession and to inform appropriate public policy responses.
